# Oxidative stress-related markers as prognostic factors for patients with primary sclerosing cholangitis in Japan

**DOI:** 10.1007/s12072-023-10557-2

**Published:** 2023-07-26

**Authors:** Atsushi Oyama, Akinobu Takaki, Takuya Adachi, Nozomu Wada, Yasuto Takeuchi, Hideki Onishi, Hidenori Shiraha, Hiroyuki Okada, Motoyuki Otsuka

**Affiliations:** Department of Gastroenterology and Hepatology, Okayama University, Faculty of Medicine, Dentistry, and Pharmaceutical Sciences, 2-5-1 Shikata-cho, Kita-ku, Okayama, 700-8558 Japan

**Keywords:** Primary sclerosing cholangitis, Oxidative stress marker, Prognosis, Serum reactive oxygen metabolite, Total serum antioxidant capacity, Revised Mayo risk score, Child–Pugh score, MELD score, FIB-4 index, Serum dROM, Serum OXY-adsorbent test, Immunoglobulin A

## Abstract

**Background/purpose:**

Primary sclerosing cholangitis (PSC) is a rare chronic liver disease. The mechanisms and prediction of PSC progression are unclear. Recent investigations have shown that general conditions, such as oxidative stress, affect the course of chronic diseases. We investigated the clinical course and oxidative stress-related condition of PSC to determine prognostic factors.

**Methods:**

We recruited 58 patients with PSC (mean age; 37.4 years, mean observation period; 1382 days) who visited our department from 2003 to 2021. Clinical characteristics were investigated to define prognostic factors. Oxidative stress status was evaluated using two types of markers: an oxidative stress marker (serum reactive oxygen metabolite; dROM) and an antioxidant marker (serum OXY adsorbent test; OXY).

**Results:**

The revised Mayo risk, Child–Pugh, model for end-stage liver disease-sodium (MELD-Na) scores or fibrosis-related FIB-4 index significantly predicted poor overall survival. High intestinal immunoglobulin A (IgA) levels predicted poor survival. Among patients with high and intermediate revised Mayo risk scores, those with physiologically high dROM levels showed better survival than those with lower dROM levels. In this population, dROM was negatively correlated with AST and IgA, which are both correlated with survival.

**Conclusions:**

High and intermediate revised Mayo risk score group predicted a poor clinical course in PSC. Additionally, the Child–Pugh score, MELD-Na score, FIB-4 index, and serum IgA were significantly correlated with survival. In patients with high and intermediate revised Mayo risk scores, physiologically high oxidative stress status correlated with low IgA levels and a good prognosis.

**Supplementary Information:**

The online version contains supplementary material available at 10.1007/s12072-023-10557-2.

## Introduction

Primary sclerosing cholangitis (PSC) is a chronic liver disease with inflammation and fibrosis of the bile ducts. Although PSC has been suggested to be an immune-related disease, the precise mechanisms have not been clearly demonstrated [1]. PSC is often concomitant with inflammatory bowel disease (IBD) and progresses to cirrhosis or hepatocellular carcinoma (HCC). PSC can also progress to colon and biliary tract cancers. PSC is a progressive disease, and therefore, risk stratification is necessary. Several prognostic models are available for predicting patient survival [1]. The revised Mayo risk score comprising age, bilirubin, albumin, aspartate aminotransferase (AST), and variceal bleeding is one of the most widely used scores that has been reported from a multi-center large cohort (*n = *405) and validated in independent cohort (*n = *124) data [2]. Liver histology assessment reported that advanced stages of the Nakanuma staging system comprising liver fibrosis, bile duct loss, and Orcein-positive chronic cholestasis could predict PSC-related death, liver transplantation, and liver-related events [3]. In addition, the fibrosis stages predicted by non-invasive imaging, such as ultrasound using transient elastography [4] or magnetic resonance imaging (MRI) using MR elastography [5], have also been valuable for assessing clinical outcomes. However, the European and United States (US) study cohorts have different clinical characteristics from those of Japanese patients with PSC. Prognosis prediction is difficult in Japanese patients because the number of patients with PSC in Japan is nearly 10 times lower than that in northern Europe or the US [6], in addition to the marked differences in clinical characteristics. The frequency of IBD as a complication is also lower in Japanese patients than in patients in Europe or the US (34–37 and 66%, respectively) [7]. Predictive models for Japanese patients with PSC are necessary to unveil the different clinical characteristics of this population.

Genes related to the immune response, such as human leukocyte antigen, have been shown to be involved in disease development in a genome-wide association study (GWAS) [8]. This relationship demonstrates that immune and inflammatory responses are related to disease progression. Immune-related liver inflammation has been shown to be correlated with oxidative stress [9]. Several approaches to define oxidative stress-related status exist. Serum levels of reactive oxygen metabolites (ROMs) are reliable markers of circulating reactive oxygen species (ROS). A dROM test that reflects ROM status has been used as an oxidative stress-related marker in several clinical conditions [10]. The total serum antioxidant capacity could be assessed by an OXY-adsorbent test (OXY), which measures the neutralizing activity of samples against hypochlorite (HClO), a powerful ROS. We have previously shown that dROM an oxidative stress-related marker, was higher in patients with chronic hepatitis (CH)-C than in those with CH-B [11]. In patients with non-alcoholic fatty liver disease (NAFLD), dROM is positively correlated with histological grade and inflammatory scores [12]. Total serum anti-oxidant levels, as assessed by OXY, were diminished in patients with HCV-related hepatocellular carcinoma.

The objective of the present study was to investigate whether the revised Mayo risk score, Child–Pugh score, model for end-stage liver disease-sodium (MELD-Na) score, and FIB-4 index predict the clinical outcome of Japanese patients with PSC and assess whether oxidative stress-related markers correlate with PSC status.

## Materials and methods

### Patients

Fifty-eight patients with PSC were followed in our hospital from June 2003 to May 2021. PSC diagnosis was confirmed through biopsy or detection of characteristic biliary tree features using magnetic resonance imaging (MRI), including MRI cholangiopancreatography (MRCP) and/or endoscopic retrograde cholangiopancreatography (ERCP). The characteristics of the study participants are summarized in Table 1. Patient condition was estimated using the revised Mayo risk score, Child–Pugh score, MELD-Na score, and FIB-4 index. The revised Mayo risk score was calculated using the following formula: *R* = 0.03 (age [y] + 0.54 ln (bilirubin [mg/dL]) + 0.54 ln (aspartate aminotransferase [U/L]) + 1.24 (variceal bleeding [0/1])–0.84 (albumin [g/dL]). The revised Mayo score was divided into three categories (high, intermediate, and low) based on the original data of the score definition [2]. The MELD score was calculated using the formula: 9.57 × ln (creatinine [mg/dl]) + 3.78 × ln (bilirubin [mg/dL]) + 11.20 × ln (INR) + 6.43. The MELD-Na score was calculated using the following formula: MELD + [1.32 × (137-Na)]˗[0.033 × MELD × (137-Na)]. The FIB-4 index was calculated using the formula ([AST × age]/[platelet count × √ALT]). Although the Child–Pugh and MELD-Na scores are scoring systems developed for liver cirrhosis, we used them to divide the patients according to decompensation.

**Table 1:** Baseline clinical characteristics of primary sclerosing cholangitis patients

	*n = *58
Follow up periods (day)	1382 (20–4605)
Age, year	37.4 (13–76)
Male sex (%)	66
Hemoglobin (g/dl)	12.5 (6.6–16.2)
Platelet (× 10^4^/μl)	29.9 (7.4–74.3)
Albumin (g/dl)	3.8 (1.5–5.2)
T. Bil (mg/dl)	3.3 (0.3–19.8)
PT-INR	1.07 (0.8–3.29)
AST (U/l)	74 (17–204)
ALT (U/l)	92 (10–475)
ALP (U/l)	884 (116–3121)
IgA (mg/dl)	308.9 (145.1–1081)
IgG4 (mg/dl) (*n = *10)	78.0 (21.7–143.84)
CA19-9 (U/ml)	395 (0.9–6478)
Revised Mayo risk score	0.058 (− 2.2–4.6)
Revised Mayo risk score (low/intermediate/high)	27/24/7
Child–Pugh score*	5 (5–13)*
Child–Pugh score (A/B/C)	40/15/3
MELD-Na score	5.6 (− 7.6–29.3)
FIB-4 index	0.78 (0.12–13.4)
FIB-4 index (< 2.67 / ≥ 2.67)	7/51
Complication with inflammatory bowel diseases	31 (53%)
Complication with bile duct cancer	6 (10%)
Liver transplant recipient candidates	8 (14%)
dROM (CARR U)	321.8 (180–471)
OXY (μmol HClO/mL)	325.1 (195–539)

Numeric data are shown as mean (range)/*median

*T.Bil* total bilirubin, *PT-INR* prothrombin time international ratio, *AST* aspartate aminotransferase, *ALT* alanine aminotransferase, *ALP* alkaline phosphatase

To estimate oxidative stress markers in other chronic liver diseases, we measured oxidative stress markers in CH-C, CH-B complicated with hepatocellular carcinoma (HCC-B), and CH-C complicated with hepatocellular carcinoma (HCC-C). CH-B was diagnosed as persistence of the hepatitis B surface antigen for > 6 months. CH-C was diagnosed as anti-hepatitis C virus antibody positive chronic liver disease. HCC was diagnosed based on radiological evidence obtained using three-phase computed tomography (CT) or MRI. Patient characteristics are shown in Table 2.

**Table 2:** Clinical characteristics of chronic liver diseases patients for oxidative stress marker analysis

	HCC-B	CH-C	HCC-C
*n*	52	29	64
Age	60 (40–84)	61 (36–83)	72 (40–88)
Male sex (%)	75	45	77
Albumin (g/dl)	4.1 (2.6–5)	4.2 (3.5–4.8)	3.5 (2.4–4.8)
Platelet (/μl)	13.7 (2.7–26.9)	18.5 (8.0–36.4)	9.4 (4.1–34.9)
T. Bil (mg/dl)	0.93 (0.42–2.62)	0.79 (0.45–1.69)	0.83 (0.30–7.44)
ALT (U/l)	26 (14–89)	30 (7–843)	37 (9–221)

MNumeric data are shown as mean (range)

*HCC-B* CH-B complication with hepatocellular carcinoma, *CH-C* chronic hepatitis C, *HCC-C* CH-C complication with hepatocellular carcinoma, T. Bil: total bilirubin, *ALT* alanine aminotransferase

Informed consent was obtained from each patient included in the study. The study protocol conformed to the ethical guidelines of the 1975 Declaration of Helsinki and was approved by the Ethics Committee of Okayama University Hospital (Approved number; Ken1603-025).

### Blood sample collection and preparation

Fasting blood samples were collected from the patients at the start of the experiment. The serum was collected on the day after admission or in the out-patient clinic, meaning that no intervention was performed before sample collection. The serum aliquots were stored at − 30 °C until the analysis was performed.

### Measurement of the serum oxidative stress related markers

Serum dROM levels were measured using a spectrophotometer (Diacron International, Grosseto, Italy). The measurement unit was the Carratelli unit (CARR U), where 1 CARR U corresponded to 0.08 mg/dL hydrogen peroxide. To determine total serum antioxidant capacity, OXY was measured using a spectrophotometer (Diacron International). This test evaluates the capacity of the serum to prevent massive oxidative activity in an HClO solution. The total antioxidant capacity was expressed in terms of the HClO (μmol) consumed by 1 mL of the sample (μmol HClO/mL).

### Statistical analysis

Statistical comparisons were performed using JMP version 14.0.0 (SAS Institute, Cary, NC, USA). Background clinical characteristics were compared using the Wilcoxon rank-sum test or Fisher’s exact test. Overall survival was calculated from the date of admission to the date of death, and survival curves were constructed using the Kaplan–Meier method. Univariate log-rank analyses were performed to determine the parameters predicting survival. Using the factors selected from the univariate analyses, multivariate Cox regression analysis was adopted to define the survival-related factors. Spearman’s rank correlation was used to evaluate the relationship between oxidative stress markers and clinical parameters. Statistical significance was set at *p < *0.05.

## Results

### Clinical characteristics of the patients

The clinical characteristics of the patients with PSC are shown in Table 1. The mean follow-up period for all 58 patients was 1382 days (range 20 − 4605 days). Eight patients died during this period. Two of the eight patients died of liver failure, and six patients died of bile duct cancer. Eight patients underwent liver transplantation, and our study defined these patients as dead at transplantation, indicating organ death. Immunoglobulin G4 (IgG4) was measured in only 10 cases (mean 78.0 mg/dl). The mean values of oxidative stress markers were dROM 321.8 CARR U and OXY 325.1 μmol HClO/mL, which were similar to the data from healthy volunteers in our previous report (dROM 306, OXY 311) [12]. Given that the clinical course, laboratory findings and oxidative stress-related status differed in the patients who died of liver failure and bile duct cancer, we conducted the same analysis with the patients without bile duct cancer (Supplemental Table 1).

### Clinical course of the patients

**Fig. 1:**
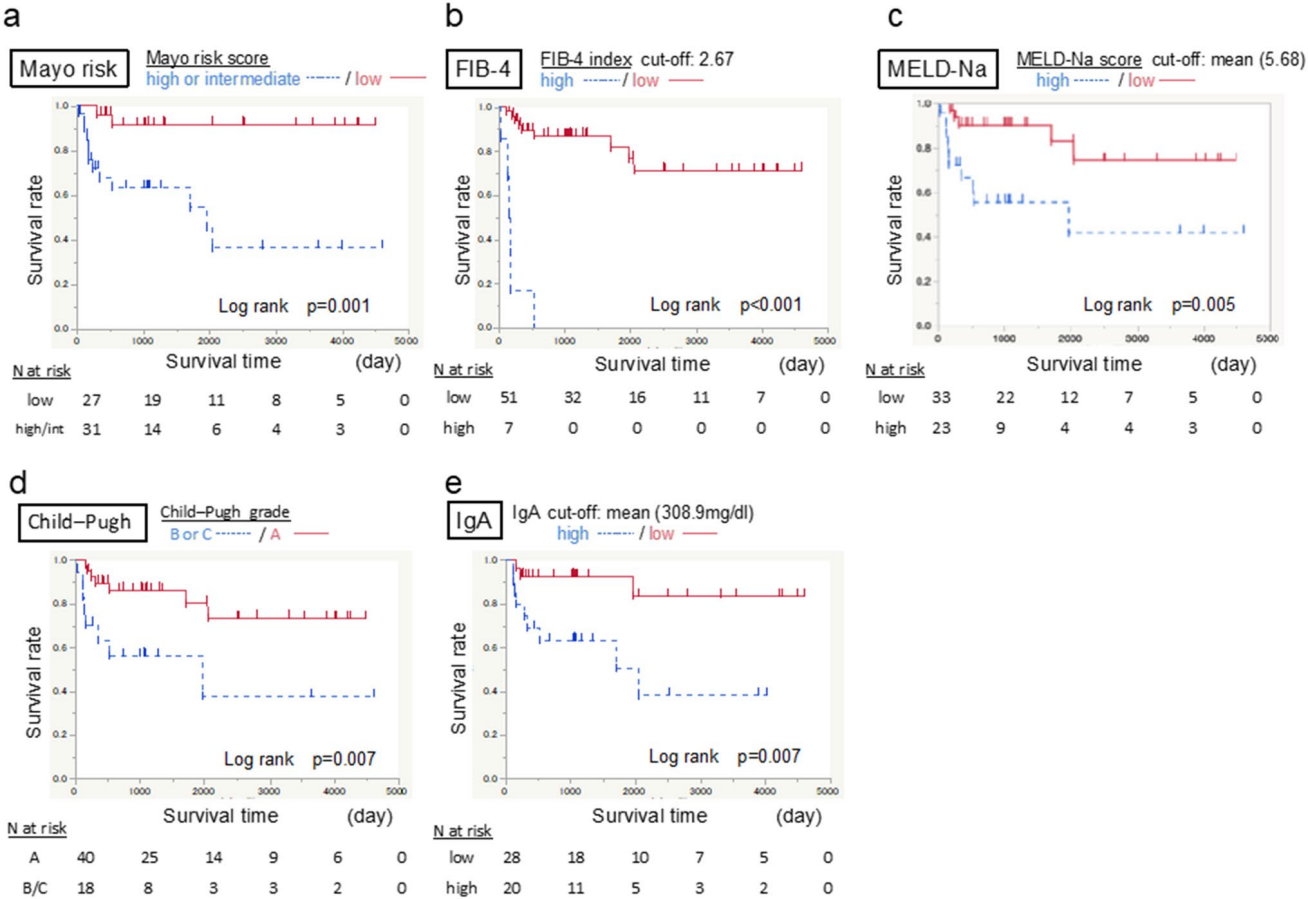
Survival rate according to clinical scores and a single marker IgA. Kaplan–Meier survival plot of the included patients. **a** Survival rates stratified according to the revised Mayo risk scores. The data demonstrate a significantly higher survival rate in patients with a low score. **b** Survival rates stratified according to the FIB-4 index. The data show a significantly higher survival rate in patients with low titers. **c** Survival rates stratified according to the MELD-Na score. The data show a significantly higher survival rate in patients with low titers. **d** Survival rates stratified according to the Child–Pugh score. The data show a significantly higher survival rate in patients with score A. **e** Survival rates stratified according to a single marker, serum immunoglobulin A (IgA). The data show a significantly higher survival rate in patients with low concentrations

The clinical course of the patients with PSC was compared in terms of the revised Mayo risk score, FIB-4 index, MELD-Na score, and Child–Pugh score (Fig. 1). These scores predicted favorable and poor patient outcomes. Patients with low Mayo risk scores had good survival rates (*p = *0.001) (Fig. 1a). Patients with low FIB-4 index (*p < *0.001) (Fig. 1b), MELD-Na score (*p = *0.005) (Fig. 1c), and Child–Pugh score (*p = *0.007) (Fig. 1d) also showed good survival. Immunoglobulin A (IgA) was not included in the above scores, however, it was an independent significant marker in predicting survival (*p = *0.007) (Fig. 1e). From our laboratory data, multivariate Cox regression analysis revealed IgA to be the only survival-related marker (Table 3). Patients with low serum IgA levels showed good survival (*p = *0.008).

**Table 3:** Univariate and multivariate analysis for the survival related laboratory data

	Log rank test	Cox logistic regression analysis		
	*p*	*p*	Hazard ratio	95% Confidence interval
Age (> 34.5 y)	0.022*	0.064	0.15	0.015–1.114
Hemoglobin (> 12.5 g/dl)	0.010*	0.918	1.11	0.157–11.135
Albumin (> 3.8 g/dl)	< 0.001*	0.342	3.17	0.303–44.355
AST (> 74 U/L)	0.012*	0.533	0.58	0.095–3.353
T.Bil (> 3.3 mg/dl)	< 0.001*	0.161	0.27	0.040–1.663
PT-INR (> 1.07)	0.008*	0.061	0.18	0.022–1.083
IgA (> 308.9 mg/dl)	0.006*	0.008*	0.13	0.022–0.611
CA19-9 (395 mg/dl)	< 0.001*	0.171	0.13	0.005–2.429

*AST* aspartate aminotransferase, *T.Bil* total bilirubin, *PT-INR* prothrombin time international ratio

*p < 0.05 by Spearman’s rank correlation coefficient

Given that the FIB-4 index is affected by aging, we compared survival according to the FIB-4 index in older and younger patient groups and found significant differences in both groups (Supplemental Fig. 1).

We conducted the same analysis with patients without complicating bile duct cancer (Supplemental Fig. 2). The difference was also significant in this subgroup.

**Fig. 2:**
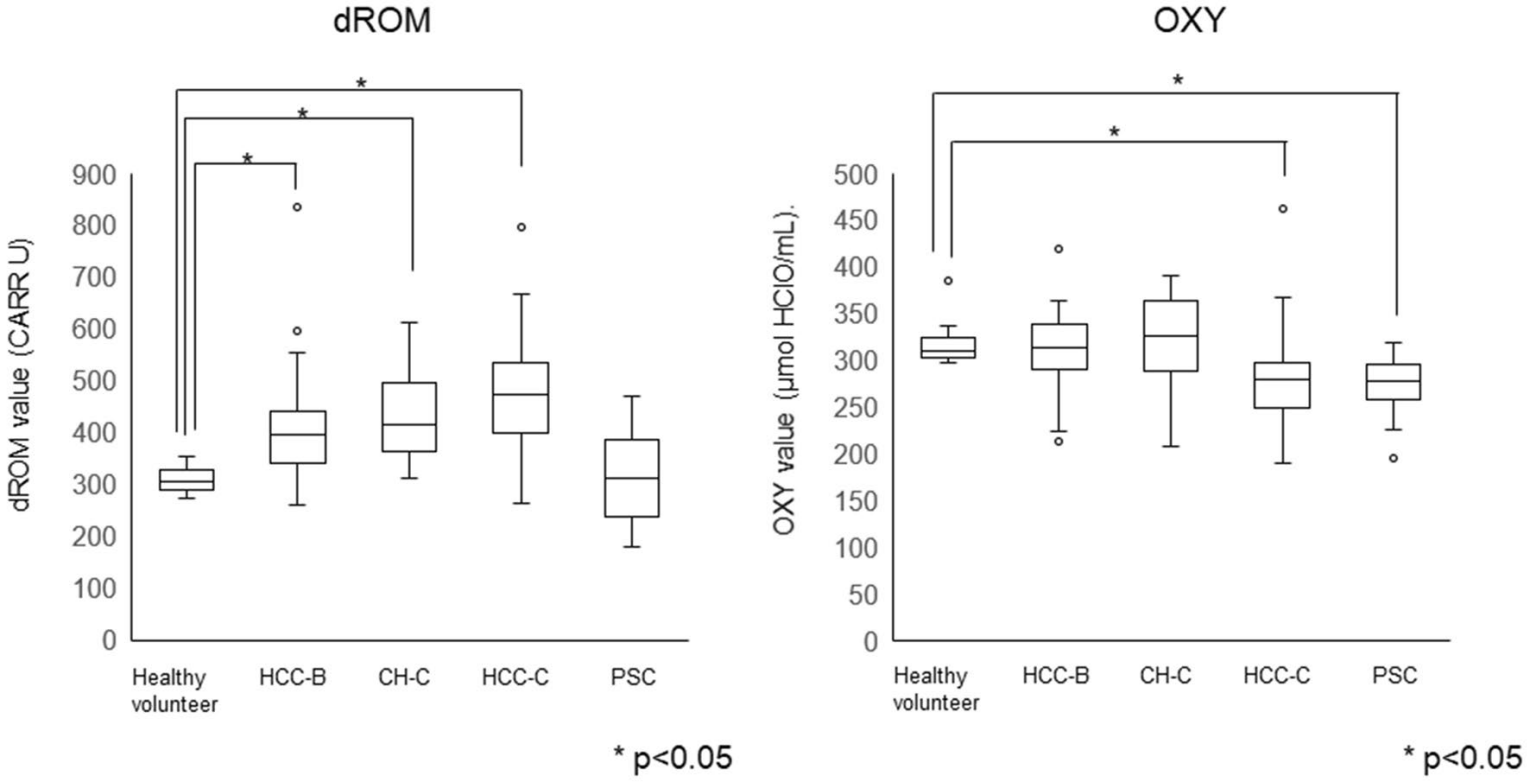
Distribution of oxidative stress-related markers, dROM and OXY, in chronic liver diseases. An oxidative stress marker (dROM) and an antioxidant marker (OXY) were measured in chronic liver diseases and compared with data from healthy volunteers (*p < 0.05). **a** The dROM was high in type B hepatocellular carcinoma (HCC-B), chronic hepatitis C (CH-C), and type C hepatocellular carcinoma (HCC-C). In patients with PSC, the dROM was not higher than that in healthy volunteers. **b** OXY was low in HCC-C and PSC

### Oxidative stress related status in chronic liver diseases

The oxidative stress-related marker, dROM, in patients with PSC was comparable to that in healthy volunteers, while patients with CH-C and HCC showed higher dROM levels than healthy volunteers (Fig. 2). The anti-oxidative status marker, OXY, was lower in patients with PSC than in healthy volunteers as well as in patients with HCC-C.

### Oxidative stress-related markers in the clinical course of PSC patients

**Fig. 3:**
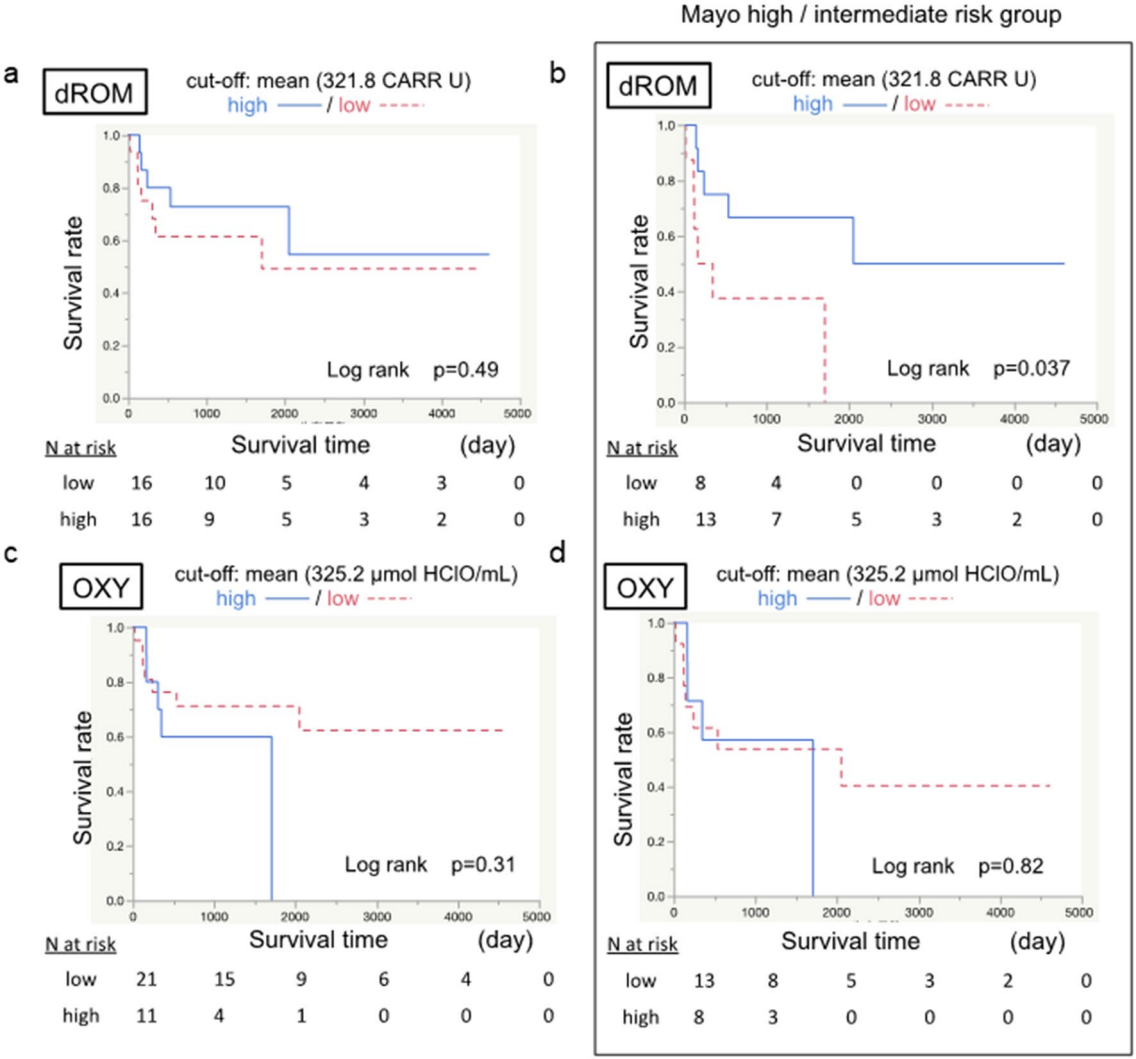
Effect of oxidative stress-related markers on the survival rate. The effects of dROM and OXY on survival rate were analyzed. **a** The overall survival rate stratified according to dROM (cut-off 321.8 CARR U) showed no difference. **b** The survival rate stratified according to dROM in the high and intermediate revised Mayo risk score group. High dROM was correlated with better survival. **c** The overall survival rate stratification according to OXY (cut-off 325.2 μmol HClO/mL) shows no difference. **d** The survival rate stratification according to OXY in the high and intermediate revised Mayo risk score group shows no difference

In patients with PSC, dROM and OXY did not correlate with survival (Fig. 3a, c). However, in patients with high and intermediate Mayo risk scores, high dROM (> mean 321.8 CARR U) predicted better survival than low dROM (Fig. 3b). Such a correlation was not found for OXY (Fig. 3d).

In the patients without complicating bile duct cancer (Supplemental Fig. 2), the data showed almost the same pattern as that of the whole cohort shown in Fig. 1. In the high and intermediate Mayo risk score group, patients with high dROM tended to show better survival, although the difference was not significant (*p = *0.057) (Supplemental Fig. 3).

### Correlation of oxidative stress related markers and the clinical characteristics of the PSC patients

To reveal which clinical characteristics affect the oxidative stress-related condition, we investigated the correlation of oxidative stress-related markers, dROM and OXY, and the patients’ clinical characteristics. In all patients with PSC, dROM was negatively correlated with IgA and positively correlated with CA19-9. OXY was not correlated with the clinical data (Table 4).

**Table 4:** Correlation between oxidative stress markers and clinical data of PSC patients

	dROM		OXY	
	Spearman’s rho	*p* value	Spearman’s rho	*p* value
Age	0.243	0.18	− 0.131	0.476
Platelet	0.205	0.260	0.134	0.465
Albumin	− 0.175	0.337	0.294	0.103
T. Bil	0.044	0.811	− 0.178	0.330
PT-INR	0.242	0.183	0.117	0.525
AST	− 0.037	0.843	0.085	0.642
ALT	− 0.070	0.706	0.206	0.258
ALP	0.192	0.293	0.116	0.527
IgG	− 0.114	0.560	− 0.096	0.625
IgM	− 0.076	0.698	− 0.167	0.394
IgA	− 0.395	0.037*	0.022	0.909
Child–Pugh	0.086	0.638	− 0.232	0.201
Mayo risk score	0.220	0.227	− 0.134	0.476
FIB-4	0.100	0.586	− 0.158	0.388
CA19-9	0.369	0.041*	0.158	0.395

*Bil* total bilirubin, *PT-INR* prothrombin time international ratio, *AST* aspartate aminotransferase, *ALT* alanine aminotransferase, *ALP* alkaline phosphatase

*Bold values indicate *p* < 0.05 by Spearman’s rank correlation coefficient

In patients with high and intermediate Mayo risk score group, where higher dROM correlated with better survival, dROM was negatively correlated with AST and IgA levels, suggesting a relationship with the intestinal immune system (Table 5). OXY was positively correlated with albumin and negatively correlated with the Child–Pugh score, suggesting a correlation between antioxidant power and liver reservoir function in this population.

**Table 5:** Correlation between oxidative stress markers and clinical data of PSC patients with Mayo risk score high and intermediate

	dROM		OXY	
	Spearman's rho	*p* value	Spearman's rho	*p* value
Age	0.081	0.724	− 0.261	0.251
Platelet (/μl)	0.257	0.260	0.174	0.450
Albumin (g/dl)	0.076	0.743	0.584	0.005*
T. Bil (mg/dl)	− 0.149	0.518	− 0.300	0.186
PT-INR	− 0.266	0.243	− 0.268	0.238
AST (U/l)	− 0.517	0.016*	0.217	0.343
ALT (U/l)	− 0.122	0.598	0.459	0.036*
ALP	− 0.016	0.942	0.200	0.384
IgG	− 0.299	0.243	− 0.252	0.328
IgM	− 0.110	0.673	− 0.321	0.208
IgA	− 0.801	< 0.001*	− 0.085	0.743
Child–Pugh	− 0.157	0.494	− 0.452	0.039*
Mayo risk score	− 0.201	0.381	− 0.310	0.170
FIB-4	− 0.254	0.265	− 0.254	0.265
CA19-9	− 0.030	0.899	0.258	0.270

*Bold values indicate *p* < 0.05 by Spearman’s rank correlation coefficient

## Discussion

In this study, scores combining known clinical characteristics, such as the revised Mayo risk, MELD-Na, and Child–Pugh scores, clearly differentiated the clinical course of patients with PSC. The liver fibrosis-related FIB-4 index and single intestinal immunoglobulin-related IgA also showed significant differences. Although oxidative stress markers did not show any differences in all outcomes of patients with PSC, high dROM was predictive of good survival in patients with high and intermediate revised Mayo scores. In this population group, dROM was negatively correlated with AST and IgA levels, both of which were correlated with survival.

The clinical course of PSC varies. There are several clinical scores predictive of survival, such as the revised Mayo, Child–Pugh, MELD, King’s College, time dependent, and PSC scores. [1]. In Japan, a nationwide survey data showed a 3-year transplant-free survival rate of 77.3%, while IgG4-related sclerosing cholangitis (IgG4-SC) has been shown to have better survival than PSC [7]. We were able to collect serum IgG4 for only 10 cases; however, we diagnosed these cases as PSC according to MRCP and/or ERCP findings. Predictive factors for survival in PSC have not been identified in these nationwide surveys. Our present data suggest that these scores could also be used for Japanese patients, although their clinical characteristics are different from those of patients in Europe and the US.

IgA comprises monomeric IgA1 and polymeric IgA2. Serum IgA is mainly composed of IgA1, while intestinal secretory IgA is mainly IgA2 [13]. Secretory IgA2 protects against potentially harmful pathogens [14]. Serum IgA level has not been shown to be a predictive factor for PSC in previous studies [15, 16], whereas our present data indicated that high serum IgA was predictive of poor survival. The significance of the measured serum IgA in the present analysis is not clear. However, several IgA antibodies have been reported to be predictive of PSC prognosis [17]. Recently, anti-pancreatic glycoprotein 2 (GP2) IgA was shown to be a candidate marker for predicting a progressive disease course in PSC [18]. GP2 is secreted from pancreatic acinar cells into the lumen and is expressed on the luminal surface of microfold cells in Peyer’s patches [19]. The anchored form of GP2 has been shown to interact with FimH + bacteria resulting in their trans-cytosis through the microfold cells, which in turn results in CD4-positive T-cell activation. These sensitized T cells can migrate to the gut and liver and induce inflammation. Another IgA autoantibody against F-actin has been reported to be associated with a strong mucosal immune response to microbial antigens resulting in progressive PSC [20]. These antibodies are mainly IgA2 detectable in the intestine and not serum IgA1. However, serum IgA antibodies have been shown to be pathogenic in IgA nephropathy in chronic kidney disease [21]. Intestinal diseases, such as celiac disease, have also been shown to contain serum IgA autoantibodies [22]. Our present data indicating that high serum IgA predicts poor prognosis suggest that disease progression-related IgA-type autoantibodies are included in serum IgA. Based on the present analysis, we lack data on the IgA subclass or autoantibody targets. This should be the next step in the investigation.

Intestinal microbiota dysbiosis is an established characteristic of chronic liver diseases and IBD, such as ulcerative colitis [23]. Gut microbiome analysis of PSC revealed high levels of *Klebsiella pneumonia.* Gnotobiotic mice inoculated with PSC-derived microbiota exhibit inflammatory T-helper 17 cell responses in the liver and increased hepatobiliary injury [24]. Oxidative stress has been shown to induce intestinal inflammatory responses resulting in intestinal microbiome composition and function changes [25]. However, physiological oxidative stress is a natural response to a toxic microbiome and is required for the plasma membrane repair response [26]. Intestinal barrier function is essential to guard the membrane and tight junctions and stop invasion by toxic microbiomes, thereby reducing intestinal inflammation [27]. Inflammation-related inflammasome-deficient mice have been shown to change their microbiota, exacerbating hepatic inflammation through an influx of bacteria related to Toll-like receptor 4 and 9 agonists into the portal circulation. This results in hepatic tumor necrosis factor (TNF)-alpha expression [28]. These findings indicate that adequate oxidative stress is necessary for controlling toxic microbiota or related molecules, although excessive stress is harmful.

Oxidative stress is involved in many chronic inflammatory diseases, including chronic hepatitis C and B and non-alcoholic steatohepatitis [9]. In previous reports on chronic viral hepatitis complicated with hepatocellular carcinoma, high dROM correlated with hepatitis C virus positivity and low albumin levels, which is a representative marker of decompensation of chronic liver disease [11]; however, in patients with NAFLD, no correlation was found with decompensation markers of chronic liver disease [12]. In patients with NAFLD, dROM is positively correlated with body mass index, hemoglobin A1c, C-reactive protein (CRP), and ferritin, which are representative of glucose homeostasis or general inflammation. These data suggest that dROM reflects oxidative stress-related conditions but does not directly reflect liver decompensation. In this study, physiologically high dROM predicted good survival in the advanced stage of PSC in patients with high and intermediate Mayo risk score. In these patients, liver inflammation-related AST and intestinal immune-related immunoglobulin IgA levels were negatively correlated with dROM. Low AST reflects low liver inflammation, and low IgA might indicate non-active intestinal inflammation or a decrease in IgA autoantibody secretion. Physiological levels of dROM-related oxidative stress are probably required to regulate liver and intestinal immune cell activation.

Adequate control of oxidative stress is necessary to manage several chronic liver diseases. However, data on toxic and adequate levels of oxidative stress are lacking. In the present analysis, a dROM level > mean of 321.8 CARR U was associated with better outcomes in patients with high and intermediate Mayo risk scores. However, this does not mean that extremely high oxidative stress is beneficial for these patients. The highest dROM in our present data was 471 CARR U, which was lower than the mean levels in our present data for patients with C-HCC (475 CARR U). Many tools are available for measuring oxidative stress conditions, and much data exist on its relation to intestinal or chronic liver disease activity [11, 29]. However, toxic levels are difficult to define, probably because the effects on each patient differ and the data often show overlap between patients with good and bad outcomes, as in our present data. Chronic inflammation in chronic liver diseases or inflammatory bowel disease was reduced with antioxidant treatment. However, antioxidant intervention often results in poor survival outcomes. In addition, attempts to regulate chronic diseases, such as carcinogenesis, using antioxidants often result in failure [30]. Adequate oxidative stress is required for self-defense from carcinogenesis and for regulating microbiota.

In the present study, dROM was negatively correlated with IgA and positively correlated with CA19-9 in all patients with PSC. Given that CA19-9 is a marker related to biliary congestion and bile duct cancer, high dROM is suggestive of advanced disease. However, in patients with high and intermediate revised Mayo scores, high dROM was correlated with better survival and negatively correlated with AST and IgA. The antioxidant marker OXY showed correlations with albumin, Child–Pugh score, and ALT levels in the high and intermediate Mayo score group but not in all patients. This could mean that these data were closely distributed near or within normal limits in the low-risk Mayo group. It should be emphasized that the high dROM in this population was not as high as that in patients with CH-B or CH-C and was comparable to that of healthy volunteers. These data suggest that physiologically high levels of dROM might guard intestines against the invasion of IgA autoantibody-coated microbiomes in the membrane and tight junctions.

In conclusion, the revised Mayo risk score, FIB-4 index, MELD-Na score, and Child-Pugh score could help predict the clinical course of Japanese patients with PSC. The oxidative stress marker, dROM, was negatively correlated with IgA, and relatively high levels of dROM predicted a better outcome in patients with high and intermediate revised Mayo risk score group. To stratify the risks in patients with PSC, those with high and intermediate revised Mayo risk scores with low dROM should be defined as more likely to exhibit disease progression.

### Supplementary Information

Below is the link to the electronic supplementary material.

Supplementary file1 Survival rate according to FIB-4 index in young age (<37.4 years) and old age groups. Kaplan–Meier survival plot of patients in the young and old age groups. Survival rates stratified according to FIB-4 index. The data show a significantly better survival rate in patients with low titers. (TIF 91 KB)

Supplementary file2 Survival rate according to clinical scores and a single marker IgA in patients without complicating bile duct cancer. Kaplan–Meier survival plot of patients without complicating bile duct cancer. a) Survival rates stratified according to the revised Mayo risk scores. The data demonstrate a significantly higher survival rate in patients with a low score. b) Survival rates stratified according to the FIB-4 index. The data show a significantly higher survival rate in patients with low titers. c) Survival rates stratified according to the MELD-Na score. The data show a significantly higher survival rate in patients with low titers. d) Survival rates stratified according to the Child–Pugh score. The data show a significantly higher survival rate in patients with score A. e) Survival rates stratified according to a single marker, serum immunoglobulin A (IgA). The data show a significantly higher survival rate in patients with low concentrations. (TIF 126 KB)

Supplementary file3 Effect of oxidative stress-related markers on the survival rate in patients without complicating bile duct cancer. The effects of dROM and OXY on the survival rate of patients without complicating bile duct cancer were analyzed. a) The overall survival rate stratified according to dROM (cut-off 321.8 CARR U) showed no difference. b) The survival rate stratified according to dROM in the high and intermediate revised Mayo risk score group. A high dROM tended to correlate with better survival. c) The overall survival rate stratification according to OXY (cut-off 325.2 μmol HClO/mL) showed no difference. d) The survival rate stratification according to OXY in the high and intermediate revised Mayo risk score group showed no difference.(TIF 118 KB)

Supplementary file4 (DOCX 17 KB)

## Data Availability

The data contains personal information and cannot open for other researchers.

## Declarations

### Clinical trial registration

This study is not registered for clinical trial registration. 
