# Supplementary figures and images for: Oxidative stress-related markers as prognostic factors for patients with primary sclerosing cholangitis in Japan
Source: Hepatol Int. 2023 Jul 26;17(5):1215–24. doi: 10.1007/s12072-023-10557-2 (PMC10522747; doi:10.1007/s12072-023-10557-2)

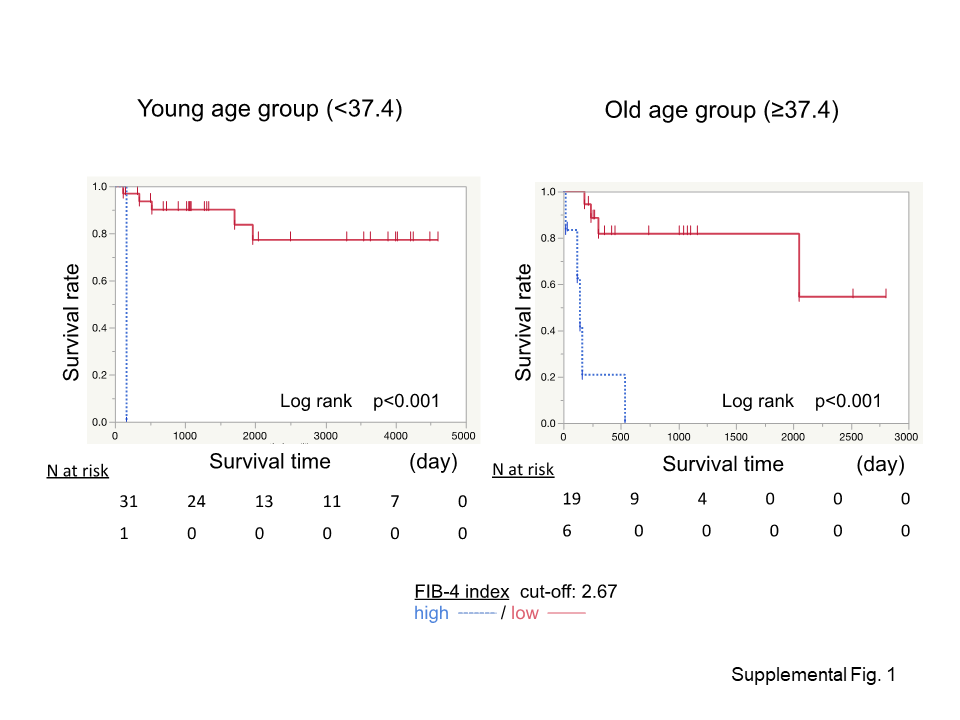

Supplement: Supplementary file 1 — Supplementary file1 Survival rate according to FIB-4 index in young age (<37.4 years) and old age groups. Kaplan–Meier survival plot of patients in the young and old age groups. Survival rates stratified according to FIB-4 index. The data show a significantly better survival rate in patients with low titers. (TIF 91 KB) [file 12072_2023_10557_MOESM1_ESM.tif]

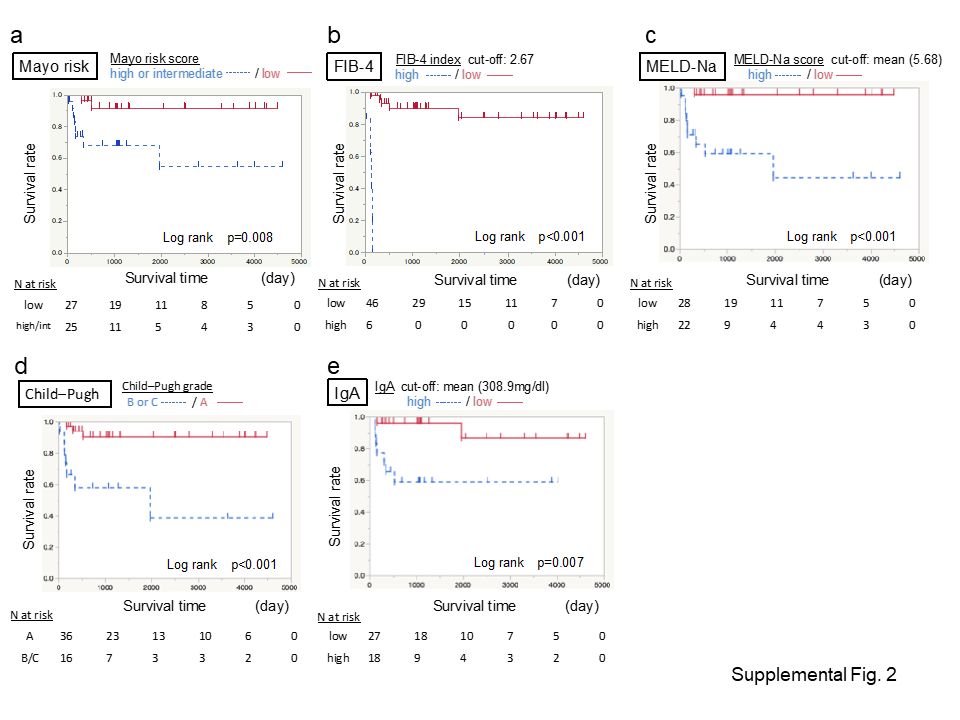

Supplement: Supplementary file 2 — Supplementary file2 Survival rate according to clinical scores and a single marker IgA in patients without complicating bile duct cancer. Kaplan–Meier survival plot of patients without complicating bile duct cancer. a) Survival rates stratified according to the revised Mayo risk scores. The data demonstrate a significantly higher survival rate in patients with a low score. b) Survival rates stratified according to the FIB-4 index. The data show a significantly higher survival rate in patients with low titers. c) Survival rates stratified according to the MELD-Na score. The data show a significantly higher survival rate in patients with low titers. d) Survival rates stratified according to the Child–Pugh score. The data show a significantly higher survival rate in patients with score A. e) Survival rates stratified according to a single marker, serum immunoglobulin A (IgA). The data show a significantly higher survival rate in patients with low concentrations. (TIF 126 KB) [file 12072_2023_10557_MOESM2_ESM.tif]

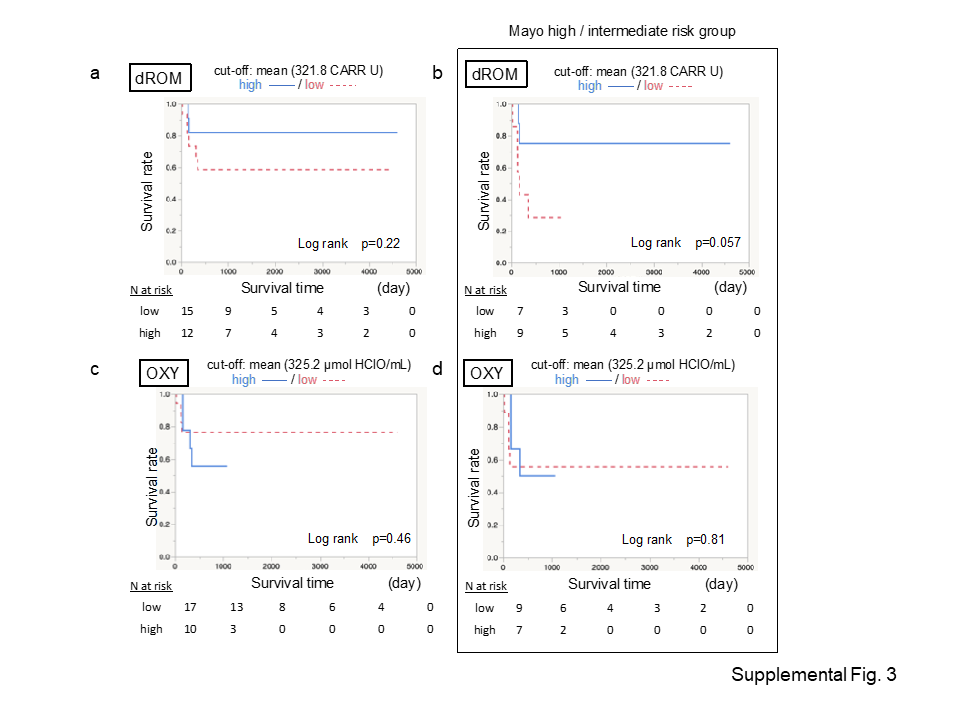

Supplement: Supplementary file 3 — Supplementary file3 Effect of oxidative stress-related markers on the survival rate in patients without complicating bile duct cancer. The effects of dROM and OXY on the survival rate of patients without complicating bile duct cancer were analyzed. a) The overall survival rate stratified according to dROM (cut-off 321.8 CARR U) showed no difference. b) The survival rate stratified according to dROM in the high and intermediate revised Mayo risk score group. A high dROM tended to correlate with better survival. c) The overall survival rate stratification according to OXY (cut-off 325.2 μmol HClO/mL) showed no difference. d) The survival rate stratification according to OXY in the high and intermediate revised Mayo risk score group showed no difference.(TIF 118 KB) [file 12072_2023_10557_MOESM3_ESM.tif]
