# Supplementary material for: Oxidative stress-related markers as prognostic factors for patients with primary sclerosing cholangitis in Japan
Source: Hepatol Int. 2023 Jul 26;17(5):1215–24. doi: 10.1007/s12072-023-10557-2 (PMC10522747; doi:10.1007/s12072-023-10557-2)
Supplement: Supplementary file 4 — Supplementary file4 (DOCX 17 KB) [file 12072_2023_10557_MOESM4_ESM.docx]

Supplemental Table 1. Baseline clinical characteristics in patients with and without complicating bile duct cancer

|  | Bile duct cancer (n=6) | No bile duct cancer (n=52) | |
| --- | --- | --- | --- |
| Follow up periods (day) | 954 (183-2060) | 1431 (20-4605) | |
| Age, year | 45.5 (30-67) | 36.5 (13-76) | |
| Male sex (%) | 66.7 | 65.4 | |
| Hemoglobin (g/dl) | 11.9 (8.9-15.4) | 12.5 (6.6-16.2) | |
| Platelet (×10^4^/μl) | 32.2 (23.0-42.1) | 29.6 (7.4-74.3) | |
| Albumin (g/dl) | 3.5 (2.7-4.3) | 3.8 (1.5-5.2) | |
| T. Bil (mg/dl) | 2.5 (0.3-8.9) | 3.36 (0.3-19.8) | |
| PT-INR | 0.96 (0.8-1.09) | 1.08 (0.83-3.29) | |
| AST (U/l) | 97 (19-204) | 72 (17-176) | |
| ALT (U/l) | 155 (10-475) | 84.9 (10-327) | |
| ALP (U/l) | 1192 (681-1873) | 849 (116-3121) | |
| IgA (mg/dl) | 353.5 (260.6-432.9) | 305.9 (145-1081) | |
| IgG4 (mg/dl) (n=10) | - | 78 (21.7-146) | |
| CA19-9 (U/ml) | 2172 (28.2-6478) | 168 (0.9-3677) | |
| Revised Mayo risk score | 0.948 (0.037-3.37) | 0.301 (-2.2-4.6) | |
| Revised Mayo risk score　(low/intermediate/high) | 0 / 5 / 1 | 27 / 19 / 6 | |
| Child-Pugh score * | 6 (5-9) | 5 (5-13) | |
| Child-Pugh score (A/B/C) | 4 / 2 / 0 | 35 / 13 / 3 | |
| MELD-Na score | 2.5 (-5.2-12.1) | 6.1 (-3.4-31.9) | |
| FIB-4 index | 1.30 (0.63-2.86) | 1.46 (0.12-13.4) | |
| FIB-4 index (<2.67 / ≥2.67) | 5 / 1 | 46/6 | |
| Complication with inflammatory bowel diseases | 3 (50%) | 28 (54%) | |
| Complication with bile duct cancer | 6 (100%) | 0 (0%) | |
| Liver transplant recipient candidates | 0 (0%) | 8 (15%) | |
| dROM (CARR U) | 371.2 (256-468) | 312.7 (180-471) | |
| OXY (μmol HClO/mL) | 308.4 (244-453) | 328.3 (195-539) | |
| Numeric data are shown as mean (range) / * as median (range)  T.Bil: total bilirubin, PT-INR: prothrombin time international ratio, AST: aspartate aminotransferase, ALT: alanine aminotransferase, ALP: alkaline phosphatase | | |  |
